# Arboviruses and COVID-19: Global Health Challenges and Human Enhancement Technologies

**DOI:** 10.3390/bioengineering12070725

**Published:** 2025-07-01

**Authors:** Rengarajan Murugesan, Srinivasan Prabhu, John T. D. Caleb, Yuvaraj Maria Francis, Indra Neel Pulidindi

**Affiliations:** 1 Department of Zoology, Annai Vailankanni Arts and Science College (Affiliated to Bharathidasan University), Thanjavur 613 007, Tamil Nadu, India; zoomurugesh@gmail.com; 2 Division of Phytochemistry and Drug Design, Department of Biosciences, Rajagiri College of Social Sciences, Cochin 683 104, Kerala, India; prabhusbotany@gmail.com; 3 Department of Anatomy, Saveetha Medical College & Hospital, Saveetha Institute of Medical and Technical Sciences Deemed University, Chennai 602 105, Tamil Nadu, India; araneae.in@gmail.com (J.T.D.C.); sujinalways@gmail.com (Y.M.F.); 4 Department of ENT, Saveetha Medical College & Hospital, Saveetha Institute of Medical and Technical Sciences Deemed University, Chennai 602 105, Tamil Nadu, India

Currently, there is increasing concern about severe illnesses, particularly those caused by viruses such as SARS-CoV-2 and arboviruses [1,2]. To date, approximately 534 species of arboviruses have been identified, of which 134 are known to affect humans. These viruses are primarily transmitted via vectors such as mosquitoes and ticks. Eventually, after entering the host, these viruses cause a range of health issues from mild to severe health complications, and even death [3]. Among arboviral infections, dengue, zika, and chikungunya have garnered significant attention from the World Health Organization (WHO) because of their profound impact on global health. However, other arboviruses remain endemic and may pose serious challenges in the near future [4]. Highlights of the major events of viral outbreaks and response milestones for arboviral diseases during the period 2023–2024 are shown in Figure 1.

Environmental factors, such as humidity and temperature, can influence the transmission of these viruses. In particular, certain arboviruses that affect animals and cause bluetongue (BT) and Schmallenberg (SB) diseases have an indirect impact on human health as well [4]. Similarly, SARS-CoV-2, the causative agent of the COVID-19 pandemic, has had an unprecedented impact on global society, economics, health, and politics. Human-to-human transmission of this virus occurs through respiratory droplets and through close contact between infected individuals. The clinical symptoms of arboviral diseases, particularly dengue fever, often closely resemble those of COVID-19, leading to frequent misdiagnoses [5]. Consequently, co-infections and overlapping symptoms of these viruses in hosts complicate diagnosis and treatment, and yet, no drugs are currently available for the management of these infections. Therefore, there is an urgent need for innovation in drug design and device fabrication to manage and reduce the health complications associated with arboviruses and COVID-19 infections [6,7].

The COVID-19 pandemic has highlighted the need for strong public health infrastructure and efficient surveillance systems. As a result, ongoing global research has focused on vector management to reduce the transmission of viruses, including COVID-19 and arboviruses, and improve resilience to future pandemics. Moreover, tackling the present and impending challenges posed by arboviruses necessitates a holistic “One Health” strategy that amalgamates both human and animal health viewpoints [5].

Vaccine development and distribution are viable public health strategies, as rapid vaccine production is feasible in response to emerging health approaches that acknowledge the interconnectedness of humans and animals. Understanding the ecological dynamics of these viruses, especially their interactions with the environment and wildlife, can help predict and prevent future outbreaks of viral pandemics. Furthermore, the strategies and technologies used to manage COVID-19, such as enhanced disease surveillance, rapid diagnostic testing, and contact tracing, can be applied to address the threat posed by arboviral infections. A comprehensive approach is essential to effectively address the significant public health threats posed by COVID-19 and arboviruses. This strategy should cover comorbid conditions, healthcare infrastructure, community impact, vaccination research, the One Health Framework, and environmental implications, along with the extensive use of gen AI-based methods. This technique is expected to prevent the transmission of these viruses and reduce the risk of future health concerns associated with them. The worldwide rise in viral diseases, especially SARS-CoV-2 and arboviruses, including dengue, Zika, and Chikungunya, has heightened public health concerns. Spread by vectors including ticks and mosquitoes, arboviruses pose major health risks. We repeat that their shared symptoms with COVID-19 cause many people to confuse the two. Although some animal arboviruses indirectly influence human health, environmental factors also help to spread them. The COVID-19 pandemic emphasized the need for robust monitoring, vector control, and a “One Health” approach that takes into account environmental, animal, and human health. Advancing vaccine research, diagnostics, and transdisciplinary strategies with the integration of gen AI tools is absolutely essential to fight these diseases and stop future epidemics caused by rising viral threats [8,9,10,11,12,13,14,15,16]. The latest breakthroughs in Human Enhancement Technologies (HETs) hold the key for bioengineering and address current global health challenges [17].

## Figures and Tables

**Figure 1:**
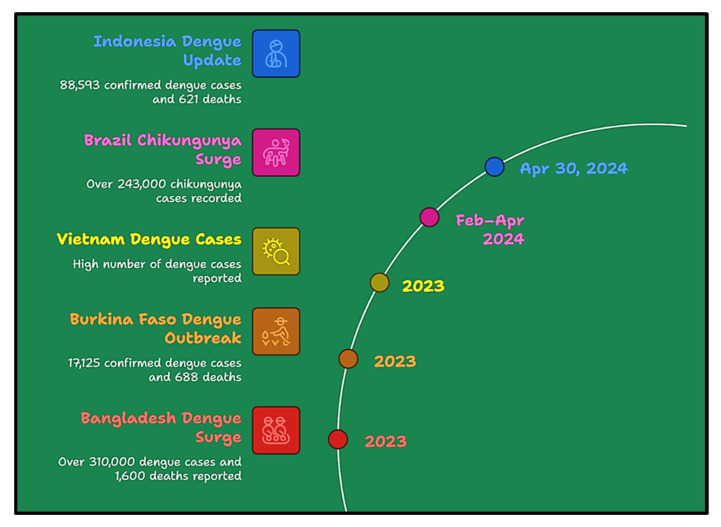
Key arboviral outbreaks during 2023–2024.
